# Performance Analysis of Cooperative Virtual MIMO Systems for Wireless Sensor Networks

**DOI:** 10.3390/s130607033

**Published:** 2013-05-28

**Authors:** Zimran Rafique, Boon-Chong Seet, Adnan Al-Anbuky

**Affiliations:** Department of Electrical and Electronic Engineering, Auckland University of Technology, Private Bag 92006, Auckland 1142, New Zealand; E-Mails: zrafique@aut.ac.nz (Z.R.); aalanbuk@aut.ac.nz (AA.-A.)

**Keywords:** cooperative virtual MIMO, wavelet based OFDM, V-BLAST, wireless sensor networks

## Abstract

Multi-Input Multi-Output (MIMO) techniques can be used to increase the data rate for a given bit error rate (BER) and transmission power. Due to the small form factor, energy and processing constraints of wireless sensor nodes, a cooperative Virtual MIMO as opposed to True MIMO system architecture is considered more feasible for wireless sensor network (WSN) applications. Virtual MIMO with Vertical-Bell Labs Layered Space-Time (V-BLAST) multiplexing architecture has been recently established to enhance WSN performance. In this paper, we further investigate the impact of different modulation techniques, and analyze for the first time, the performance of a cooperative Virtual MIMO system based on V-BLAST architecture with multi-carrier modulation techniques. Through analytical models and simulations using real hardware and environment settings, both communication and processing energy consumptions, BER, spectral efficiency, and total time delay of multiple cooperative nodes each with single antenna are evaluated. The results show that cooperative Virtual-MIMO with Binary Phase Shift Keying-Wavelet based Orthogonal Frequency Division Multiplexing (BPSK-WOFDM) modulation is a promising solution for future high data-rate and energy-efficient WSNs.

## 1. Introduction

Due to advancement in Micro-Electro-Mechanical Systems (MEMS) technology, low power and low cost WSNs can be deployed in many real life applications, including environmental monitoring, home automation, traffic control, precision agriculture and health care [1–3].Wireless multimedia sensor networks (WMSNs) [4] where sensor nodes are capable of producing different media streams (audio, video, image, textual, and scalar sensor data), are an emerging type of sensor networks which can facilitate automated real-time interpretation of situations in the monitored environment. Potential applications of such sensor networks include country borders and public spaces surveillance, wildlife habitat and seismic monitoring, in-home emergency detection for the sick and elderly, mixed reality networked gaming, and quality control of manufacturing processes [5]. However, multimedia contents such as image or video streams require data rates that are orders of magnitude higher than what can be supported by current WSNs. Embedded sensors are also constrained in terms of energy as they are typically battery-powered [6]. Thus, high data rates and high energy efficiency are key issues to be addressed in such networks.

MIMO techniques can be used to increase data rate using spatial multiplexing and bit error rate (BER) can be improved by using spatial diversity. MIMO techniques can also be used to improve signal to noise ratio (SNR) at the receiver and to mitigate co-channel interference (CCI) along with beam forming techniques [7]. However, MIMO systems also have a higher circuit complexity, which consumes energy. In long distance transmission, circuit energy consumption is typically much lower than transmission energy consumption. In short distance transmission, however, circuit energy consumption can be comparable with transmission energy consumption [8]. Thus, to evaluate the performance of MIMO techniques in energy limited WSNs, where sensors are mostly powered by batteries or other exhaustible energy sources, one must take into account of both circuit and transmission energy consumption.

In true/co-located MIMO architecture, multiple antennas are connected to a single transmitter/receiver node. This architecture can be used for space division multiplexing (SDM) as well as for space time coding (STC). The signal processing can be done at transmitter and/or receiver side. However, due to small form factor of wireless sensor nodes, limited energy availability, and the need to maintain a minimum distance among the antennas (to avoid fading), it can be difficult to realize the advantages of MIMO techniques for such wireless nodes [9]. Thus, the concept of virtual (cooperative/distributive) MIMO was explored for energy and physically constrained WSN nodes in [8] using Alamouti coding [10]. In virtual MIMO, multiple single-antenna nodes can be grouped as one entity, and each node shares its antenna with others in the group to function cooperatively as one MIMO system. To achieve almost ideal true MIMO performance, a virtual MIMO system with adaptive modulation and different source coding techniques has been proposed recently [11,12]. Virtual MIMO with V-BLAST [13] multiplexing architecture has also been explored, which showed significant energy savings as compared to traditional Single-Input Single-Output (SISO) based systems [14].

This paper focuses on cooperative virtual MIMO systems based on V-BLAST architecture for WSNs. Specifically, it analyzes the performance of such systems under different modulation techniques, including multi-carrier modulation techniques, which to our knowledge have yet to be investigated in literature for such systems. The modulation techniques considered include Fourier based OFDM (FOFDM), WOFDM, BPSK-FOFDM, BPSK-WOFDM, *M*-ary Quadrature Amplitude Modulation (QAM), *M*-ary Differential Quadrature Phase Shift Keying (DQPSK), and *M*-ary Offset Quadrature Phase Shift Keying (OQPSK). The analysis is performed across a broader range of performance metrics than previous related studies [8,11,12,14] including BER, energy efficiency, spectral efficiency, and time delay performances. Given the critical importance of energy in WSNs, the detailed modeling and analysis of communication (circuit and transmission) energy consumption and processing (CPU or central processing unit) energy consumption of WSN nodes in different operating modes, is another key contribution of this paper. Findings of this study can provide useful insights into certain performance aspects and identify promising solutions for future high data-rate and energy-efficient WSNs.

The rest of the paper is organized as follows: Section 2 gives an overview of related works on performance evaluation of MIMO systems for WSNs. Section 3 introduces background concepts on cooperative virtual MIMO, V-BLAST, and multi-carrier modulation. In Section 4, the system model of the cooperative virtual MIMO WSN is presented. This is followed by the parametric modeling of performance parameters for virtual MIMO and SISO systems in Section 5. Evaluation results are then presented and discussed in Section 6. Finally, the paper is concluded in Section 7.

## 2. Related Work

In [8], the energy and delay performances of cooperative virtual MIMO system with Alamouti coding for WSNs were investigated and compared with SISO system for the same throughput and BER. The performance was also compared over different transmission distances with the contemplation of circuit and transmission energy consumption. Alamouti coding is an STC technique in which space and time (two-dimensional coding) with multiple antenna setups can be used to attain coding gain and diversity gain for the same bit rate, transmission power and bandwidth as compared single antenna system. In STC techniques, information bits are transmitted according to some pre-defined transmission sequence. At the receiver, the received signals are combined by using optimal combining scheme followed by a decision rule for maximum likelihood detection [10].

A V-BLAST based virtual MIMO WSN with QAM was proposed in [14], which does not require spatial encoding on transmitting side nodes, thus eradicating the local communication and corresponding synchronization requirement on transmitting side nodes as previously involved in [8]. To make the system more energy efficient without any information loss, the use of WOFDM with V-BLAST based WSN was proposed in [15] and evaluated under a co-located true MIMO receiver architecture. In [16], the BER performance of such systems was also observed using different V-BLAST detection algorithms.

With the advent of smart antennas for WSNs [17,18], a non-cooperative STC technique based MIMO system was recently proposed in [19]. By using 2-element switched antenna array, there is no requirement for local communication at transmitter and receiver side which makes the system more energy efficient. To simplify the structure of MIMO WSN for energy consumption reduction, a nonlinear MIMO technique was proposed in [20], where real or imaginary part of the complex-valued received signal was considered for further processing which results in simpler receiver architecture at the cost of some information loss.

Based on existing studies, cooperative virtual MIMO with V-BLAST detection can be a promising communication architecture for WSNs. Furthermore, the choice of the modulation scheme for use with the architecture is also critical for reliable communication in WSNs. As discussed in [1], the modulation technique should be simple and low-power, and whose characteristics preferably can be tailored according to the channel conditions. To our knowledge, the performance of multi-carrier modulation techniques have not been studied in V-BLAST based virtual MIMO system for WSNs.

Multi-carrier modulation techniques such as WOFDM is promising for enabling high data-rate WSNs and which can be implemented with low complexity [21]. Unlike its counterpart, FOFDM, whose bases are static sine/cosine, wavelet bases of WOFDM can also be optimized according to system or channel requirement.

## 3. Background

### 3.1. Cooperative Virtual MIMO

MIMO techniques are capable of providing high system performance without additional transmission power and bandwidth. However, due to the small form factor and limited energy of sensor nodes, it is often not realistic to equip each sensor with multiple antennas to implement MIMO. Instead, a cluster of single-antenna sensor nodes can cooperate to form a virtual antenna array (VAA) to achieve virtual MIMO communication. Virtual MIMO systems are distributed in nature because multiple nodes are placed at different physical locations to cooperate with each other. With proper timing and frequency synchronization between constituent nodes of the VAA, virtual MIMO can realize the advantages of true MIMO techniques for WSNs.

### 3.2. V-BLAST

V-BLAST is a spatial multiplexing technique to achieve spectral efficiency for a given bit rate and transmission power. It can boost channel capacity to improve the single-sensor data rate, or increase the number of supported sensors in the system. Its spectral efficiency ranges from 20–40 bps/Hz [13] while efficiency of traditional wireless communication techniques ranges from 1–5 bps/Hz (mobile cellular) to around 10–12 bps/Hz (point to point fixed microwave system). In V-BLAST a single user's data stream is split into multiple sub-streams or multiple users can transmit their data simultaneously. An array of transmitter antennas is used to transmit all sub-streams simultaneously in the same frequency band, hence the spectrum is used very efficiently. Since the user's data is being sent in parallel over multiple antennas, the effective transmission rate is increased approximately in proportion to the number of transmit antennas used. In this system, the number of receivers is greater than or equal to the number of transmitters. The transmitted sub-streams are independent of one another. Individual transmitter power is scaled by *1*/*N_t_*. Thus, the total power remains constant independent of the number of transmitters (*N_t_*).

### 3.3. Multi-Carrier Modulation

FOFDM is a multi-carrier modulation technique in which a high data rate substream is demultiplexed into lower data rate substreams to increase the duration of each substream so that inter-symbol interference (ISI) can be reduced. The orthogonal subcarriers are generated using sine/cosine bases and the orthogonality is achieved in a time window of width equal to the duration of the symbol. Therefore, FOFDM is not band limited. Each subcarrier produces side lobes that in turn create inter-carrier interference (ICI), which can be increased due to multipath channel effect that also cause an increase in ISI. Cyclic prefix (CP)/Guard Interval (GI) is added to each FOFDM symbol to avoid this problem at the cost of transmission efficiency degradation.

**Figure 2.**
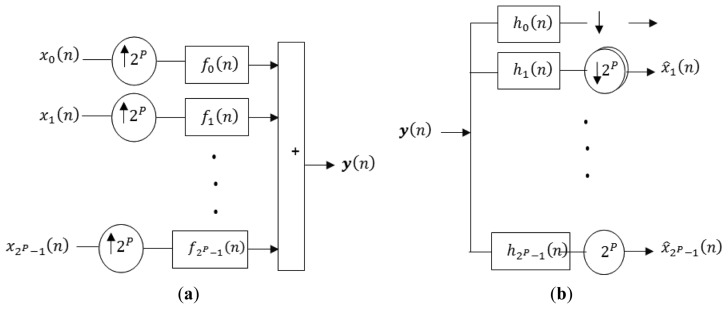
Equivalent structure of WOFDM modulator and demodulator using noble identities. (**a**) A 2*^P^* sub-channel WOFDM modulator; (**b**) A 2*^P^* sub-channel WOFDM demodulator.

WOFDM is another multi-carrier modulation technique with lower computational complexity than FOFDM [22]. This technique is also a strong candidate for high data rate communication systems [23], and therefore will be explained in more detail. The orthonormal wavelets in WOFDM can be generated using symmetric or asymmetric multistage tree structure of Quadrature Mirror Filter (QMF) bank. The symmetric multistage synthesis and analysis side QMF bank is shown in Figure l(a,b) respectively. The equivalent structure of WOFDM modulator and de-modulator using noble identities is shown in Figure 2. By using symmetric structure, the orthonormal wavelets are given by the following equation:

(1) $$f_{k}(n)=\prod_{p=1}^{P}\sqrt{2}t_{k,p}\;\left(\frac{n}{2^{p-1}}\right)$$

where ∏ represents the convolution operation, *P* is the number of levels of this structure, *k* ∈ {0,1,2,3,…, 2*^P^* − 1}, and *t_k,p_*_(*n*)_ ∈ {*f_l_*_(_*_n_*_)_, *f_h_*_(_*_n_*_)_} is the filter impulse response corresponding to *k_th_* sub-channel at *p_th_* level. *f_l_*_(_*_n_*_)_ and *f_h_*_(_*_n_*_)_ are impulse responses of the low-pass, and high-pass filters respectively, for perfect reconstruction of QMF bank. The high pass filter can be derived from the low pass filter by the relation: *f_h_*(*n*) = (−1)^n^*f_l_*(*U* − 1 − *n*), where *U* is the length of the filter [24].

The output *y*(*n*) of WOFDM modulator can be expressed as:

(2) $$y(n)=\sum_{k=0}^{2^{P}-1}\sum_{m}x_{k}(m)f_{k}(n-2^{P}m)$$

where *x_k_*(*n*) is the *k_th_* sub-channel input of WOFDM modulator. For WOFDM demodulation, the orthonormal wavelet bases are generated using symmetric analysis side QMF bank as follows:

(3) $$h_{k}(n)=\prod_{p=1}^{P}\frac{1}{\sqrt{2}}u_{k,p}\;\left(\frac{n}{2^{p-1}}\right)$$

where *u_k,p_*_(_*_n_*_)_ ∈ {*g_l_*_(_*_n_*_)_, *g_h_*_(_*_n_*_)_} is the filter impulse response corresponding to *k_th_* sub-channel at *p_th_* level, *g_l_*_(_*_n_*_)_ and *g_h_*_(_*_n_*_)_ are time reversals of *f_l_*_(_*_n_*_)_, and *f_h_*_(_*_n_*_)_, respectively [25].

**Figure 3.**
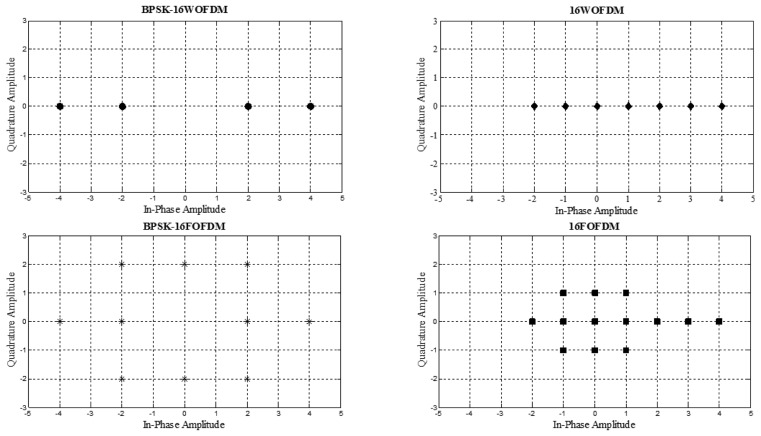
Real and imaginary components of BPSK-16WOFDM, 16WOFDM, BPSK-16FOFDM, and 16FOFDM.

From Figure 3, it can also be observed that the constellation of FOFDM and BPSK-FOFDM is two-dimensional while that of WOFDM and BPSK-WOFDM is one-dimensional. Due to this reason, RF section of FOFDM and BPSK-FOFDM as well as *M*-ary QAM, *M*-ary DQPSK and *M*-ary OQPSK which shares a similar transmitter and receiver architecture, is potentially more complex as compared to that of WOFDM and BPSK-WOFDM.

## 4. System Model

**Figure 4.**
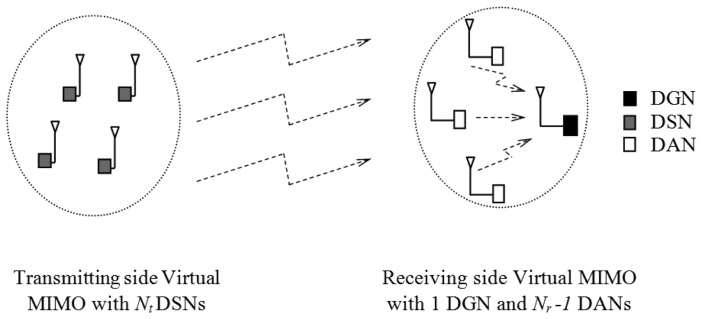
Communication between Transmitting and Receiving side Virtual MIMO nodes.

We consider a wireless communication link between *N_t_* data sensing nodes (DSNs) serving as one Virtual MIMO transmitting side node, and one Virtual MIMO receiving side node which consists of one single-antenna data gathering node (DGN) and *N_r_* − 1 data assisting nodes (DANs), each with one antenna as shown in Figure 4.

In our system model, we consider V-BLAST signal processing by DGN at the receiving side with the assumption that it can cope with more computational complexity than its DANs. Moreover, no local communication and processing are essential among the DSNs. It is assumed that *N_t_* DSNs are transmitting their data simultaneously over a flat fading MIMO channel to DGN (referred to as long-haul communications [8,14]). In addition, there are *N_r_* − 1 DANs in close proximity of DGN to form one virtual receiving node of size *N_r_*, including the DGN itself. All DANs transmit their data using time-division-multiple access (TDMA) to DGN (referred as local communication on receiving side) to form received signal vector *Rec* as shown in the following equation:

(4) Rec=HS+ζ

where *Rec* is an *N_r_* × *1* vector, *S* is *N_t_* × *1* vector, *ζ* is an *N_r_* × *1* noise vector whose elements are complex Gaussian random variables with zero mean and variance *N_o_*, *H* is an *N_r_* × *N_t_* channel matrix. It is assumed in this article that *N_t_* ≤ *N_r_* [14].

At each DSN, a serial-to-parallel converter is used to form the input for WOFDM modulator. Every *k_th_* input is first up-sampled by 2*^P^* and then filtered by sub-channel impulse response *f_k_*(*n*). Received signal vectors at DGN are detected using QR decomposition detection algorithm [26]. Denoting channel response matrix *H* = *QR*, where *Q* is *N_r_* × *N_t_* unitary matrix composed of orthonormal columns with unit norm and *R* is *N_t_* × *N_t_*, upper triangular matrix, the received signal expression in Equation (4) can be modified to detect the transmitted signals by multiplying it with *Q^t^* (transpose of *Q*) as follows:

(5) $$\tilde{\text{Rec}}=Q^{t}\text{Rec}=Q^{t}(HS+\zeta )=Q^{t}\text{QRS}+\eta$$

(6) $$\tilde{\text{Rec}}=RS+\eta$$

where *Q^t^Q* = *I* (I is identity matrix), and *η* = *Q^t^ζ* is statistically identical to *ζ*. Due to upper triangular structure of R, the *i*_th_ element of $\tilde{\text{Rec}}$ is given by:

(7) $${\tilde{\text{rec}}}_{i}=r_{ii}+d_{i}+\eta_{i}$$

where $d_{i}=\sum_{j=i+1}^{N_{t}}r_{ij}s_{i}$ is the interference term. The interference free signal element is given by:

(8) $$z_{i}={\tilde{\text{rec}}}_{i}-d_{i}$$

and the detected signal $\hat{S_{\iota }}=\frac{z_{i}}{r_{ii}}$, corresponding to each receiving antenna, is demodulated using WOFDM demodulator. The detected signal stream is first filtered by sub-channel impulse response *h_k_*(*n*) and then down-sampled by 2*^P^*. For BPSK-WOFDM system, at each DSN, the bit stream is first modulated using BPSK modulator and then fed to a serial-to-parallel converter to form the input for WOFDM modulator.

## 5. Parametric Modeling of System Characteristics

### 5.1. Energy Consumption

In [8,14], the energy consumed in baseband signal processing blocks were neglected to keep the energy consumption model simple. However, in this paper, we have also computed the energy consumed by baseband (Digital) signal processing blocks. The DGN (often a more resourceful node serving as a sink) is considered to have no energy constraints unlike the DSNs and DANs [14].

#### RF (Analog) Energy Consumption

The total energy consumption in RF section is due to long-haul communication (from DSNs to receiving side DANs and DGN itself) and receiver side local communication (from DANs to DGN). The total average power consumption along the signal path for long-haul can be divided into two main components: power consumption of all power amplifiers $P_{LA}^{L}$, and power consumption of all other circuit blocks $P_{c}^{L}$ [8]. As in [27], we assume that the power consumed by power amplifiers is linearly dependant on the transmit power $P_{\text{out}}^{L}$:

(9) $$P_{PA}^{L}=(1+\alpha )P_{\text{out}}^{L}$$

where *α* = *μ*/*ς* with *ς* being the drain efficiency of the RF power amplifier, and *μ* being the peak-to-average power ratio (PAPR) [28], which depends on the modulation scheme and associated constellation size [8]. $P_{\text{out}}^{L}$ can be calculated according to link budget relationship [29] as follows:

(10) $$P_{\text{out}}^{L}={\bar{E}}_{b}^{L}R_{b}\frac{{\left(4\pi d^{L}\right)}^{2}}{G_{t}G_{r}\lambda^{2}}M_{l}N_{f}$$

where ${\bar{E}}_{b}^{L}$ is the required energy per bit for a given BER ${\bar{P}}_{b}^{L}$ at receiver side, *R_b_* is the bit rate of the system, *d^L^* is the distance between transmitting and receiving side cluster, *G_t_* and *G_r_* are the transmitter and receiver antennas gains respectively, *λ* is the carrier wavelength, *M_l_* is the link margin for compensating the hardware process variations and other additive background noise or interference, and *N_f_* is the receiver noise figure.

**Figure 5.**
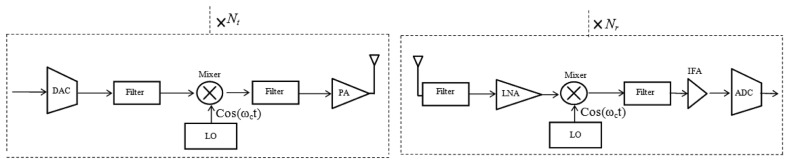
Transmitter and receiver architecture for WOFDM (analog).

The power consumption in all circuit blocks for long-haul communication with *N_t_* transmitter circuits and *N_r_* receiver circuits using WOFDM transmitter and receiver architecture as shown in Figure 5, can be calculated as:

(11) $$P_{c}^{L}\approx N_{t}[P_{\text{DAC}}+P_{\text{mix}}+(2\times P_{\text{fil}})+P_{LO}]+N_{r}[P_{\text{LNA}}+P_{\text{mix}}+(2\times P_{\text{fil}})+P_{LO}+P_{\text{IFA}}+P_{\text{ADC}}]$$

where *P_DAC_*, *P_mix_*, *P_ftl_*, *P_LO_*, *P_LNA_*, *P_IFA_* and *P_ADC_* are the power consumption values for the digital-to-analog converter (DAC), the mixer, filter, local oscillator, low-noise amplifier (LNA), intermediate-frequency amplifier (IFA) and analog-to-digital converter (ADC), respectively. In addition, the energy models developed in [27] can be used to estimate the values for *P_DAC_* and *P_ADC_*.

**Figure 6.**
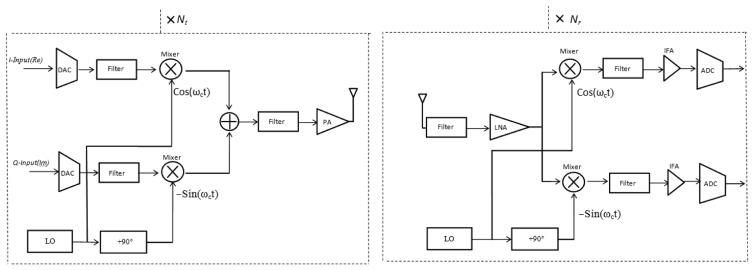
Transmitter and receiver architecture (In-Phase/Quadrature-Phase) for FOFDM, QAM, DQPSK, and OQPSK (analog).

Total power consumption in all circuit blocks for long-haul communication with *N_t_* transmitter circuits and *N_r_* receiver circuits using In-Phase/Quadrature-Phase (FOFDM and QAM) transmitter and receiver architecture as shown in Figure 6, can be calculated as:

(12) $$\begin{array}{lll} P_{c}^{L} & \approx & N_{t}[(2\times P_{\text{DAC}})+(2\times P_{\text{mix}})+(3\times P_{\text{fil}})+P_{LO}+P_{PS}+P_{\text{Add}}]\\ & & +N_{r}[P_{\text{LNA}}+(2\times P_{\text{mix}})+(3\times P_{\text{fil}})+P_{LO}+P_{PS}+(2\times P_{\text{IFA}})+(2\times P_{\text{ADC}})] \end{array}$$

where *P_PS_* and *P_Add_* are the power consumption values for phase shifter, and adder respectively. The total energy consumption per bit for long-haul communication can then be obtained as follows:

(13) $$E_{b}^{L}=\frac{P_{PA}^{L}+P_{c}^{L}}{R_{b}}$$

where *R_b_* is the data rate in bits per second (bps). The total energy consumption per bit for local communication can be obtained as follows:

(14) $$E_{b}^{l}=\frac{P_{PA}^{l}+P_{c}^{l}}{R_{bi}}$$

$P_{PA}^{l}$ is the power of each amplifier of DAN during local communication and its value can be obtained by using Equations (10) and (11) and substituting the parameters ${\bar{E}}_{b}^{L}$, *R_b_*, *G_t_*, *G_r_*, *d^L^* with ${\bar{E}}_{b}^{l}$, *R_bi_*, *G_ti_*, *G_ri_*, *d^l^*, where ${\bar{E}}_{b}^{l}$ is the required energy per bit for a given BER ${\bar{P}}_{b}^{l}$ at DGN side, *R_bi_* is the bit rate of each individual node *i*, *d^l^* (≪ *d^L^*) is the distance between DAN and DGN, *G_ti_* is the antenna gain of each DAN, *G_ri_* is the antenna gain of DGN. Circuit power consumption for local communication $P_{c}^{l}$ can be calculated using Equations (12) and (13) by replacing *N_r_* = *N_t_* = 1. The total energy per bit per node (RF section) can be calculated using the following equation:

(15) $$E_{b\_\text{Analog}}=\frac{E_{b}^{L}+(N_{r}-1)E_{b}^{l}}{N_{t}+N_{r}}$$

The energy efficiency (EE) can be calculated by taking the inverse of Equation (15).

#### Base Band (Digital) Energy Consumption

The number of CPU cycles of a processing block is estimated by using Odyssey prediction model [30]. To calculate the base band energy consumption of a block, the TelosB mote [31] energy consumption per CPU cycle [32] value is multiplied by the estimated number of CPU cycles. The energy consumed per bit by CPU during modulation (*E_Mod_*), which also represents the base band energy consumption in transmit (T_x_) mode, can be calculated by multiplying the estimated number of CPU cycles of the modulation processing block with energy consumption per CPU cycle and dividing it with total number of bits. The energy consumption per bit by CPU during demodulation (*E_Dmod_*) and V-BLAST detection (*E_Det_*) can be calculated similarly, and their sum represents the base band energy consumption in receive (R_x_) mode. With *N_t_* transmitters (DSNs) and *N_r_* receivers (DANs and DGN), the total base band energy consumption per bit per node can be calculated as:

(16) $$E_{b\_\text{Digital}}=\left(\frac{N_{t}E_{\text{Mod}}+N_{r}E_{\text{Dmod}}+E_{\text{Det}}}{N_{t}+N_{r}}\right)$$

### 5.2. Spectral Efficiency

The spectral efficiency (SE) of a MIMO system without the knowledge of the channel at the transmitter can be calculated as [33]:

(17) $$S_{E}=\sum_{j=1}^{N_{t}}\log_{2}\;\left(1+\frac{E_{b}R\gamma_{j}}{N_{o}BN_{t}}\right)$$

where *γ_j_* is the eigen value of $H_{n}H_{n}^{H}$, $H_{n}=\frac{\sqrt{N_{r}}}{{\left\|H\right\|}_{\text{Forbenius}}}H$ is the normalized channel matrix [34], and (.)*^H^* denotes Hermitian transpose.

### 5.3. Time Delay

The total time delay (*T_V_*_–_*_mimo_*) of virtual MIMO system with *N_t_* DSNs, *N_r_* − 1 DANs and one DGN each with one antenna can be calculated as the sum of transmission delay (*T_tr_MIM0_*), propagation delay (*T_pr_MIMO_*) and processing delay (*T_pc_MIMO_*):

(18) $$T_{V-\text{MIMO}}=T_{tr\_\text{MIMO}}+T_{pr\_\text{MIMO}}+T_{pc\_\text{MIMO}}$$

*T_tr_MIMO_* is given by:

(19) $$T_{tr\_\text{MIMO}}=T_{s}\left(\frac{\sum_{i=1}^{N_{t}}M_{i}}{b_{n}}+\sum_{j=1}^{N_{r}-1}\frac{m_{r}M_{s}}{b_{j}^{r}}\right)$$

where *M_i_* is the number of bits transmitted by each node *i*, $M_{s}=\sum_{i=1}^{N_{t}}\frac{M_{i}}{b_{n}}$ is the total number of symbols received, *b_n_*, $b_{j}^{r}$ are the constellation sizes (bits per symbol) used at transmitter side, and receiver side, local communication respectively, *m_r_* represents the number of bits after quantisation of each symbol received at receiver side relay nodes, $T_{s}\approx \frac{1}{B}$ is the symbol duration, and *B* is the transmission bandwidth. *T_pr_MIMO_* is given by:

(20) $$T_{pr\_M\text{IMO}}=\frac{d^{L}}{c}+\frac{d^{l}}{c}$$

where *c* is the speed of light, and *d^L^* and *d^l^* as defined after Equation (10), and Equation (14), respectively.

*T_pc_MIMO_* is given by:

(21) $$T_{pc\_\text{MIMO}}=T_{\text{Mod}}+T_{\text{Dmod}}+T_{\text{Det}}$$

where *T_Mod_*, *T_Dmod_* and *T_Det_* are the processing time values for modulator, demodulator, and detection algorithm, respectively. Each block processing time is calculated by dividing the estimated CPU cycles (as mentioned in Section 5.1.2) with TelosB mote processing speed [31].

### 5.4. SISO System

For the SISO system, the RF (Analog) energy consumption per bit per node (E_b_Analog_SISO_) can be calculated by replacing ${\text{P}}_{\text{PA}}^{\text{L}}$, ${\text{P}}_{\text{out}}^{\text{L}}$, ${\bar{\text{ E}}}_{\text{b}}^{\text{L}}$, ${\text{P}}_{\text{c}}^{\text{L}}$ with ${\text{P}}_{\text{PA}}^{\text{SISO}}$, ${\text{P}}_{\text{out}}^{\text{SISO}}$, ${\bar{\text{E}}}_{\text{b}}^{\text{SISO}}$, ${\text{P}}_{\text{c}}^{\text{SISO}}$, respectively, and also replacing N_r_ = N_t_ = 1 as discussed in Section 5.1.1. The Base Band (Digital) energy consumption per bit per node (E_b_Digital_SISO_) can be calculated by removing E_Det_ in Equation (16) and assigning N_r_ = N_t_ = 1.

The total time delay (T_SISO_) of the SISO system can be calculated as the sum of transmission delay (T_tr_SISO_), propagation delay (T_pr_SISO_), and processing delay (T_pc_SISO_).

(22) $$T_{\text{SISO}}=T_{tr\_\text{SISO}}+T_{pr\_\text{SISO}}+T_{pc\_\text{SISO}}$$

*T_tr_SISO_* is given by:

(23) $$T_{tr\_\text{SISO}}=T_{s}\frac{M_{t}}{b_{n}}$$

where *M_t_* is the total number of bits transmitted. *T_pr_SISO_* is given by:

(24) $$T_{pr\_\text{SISO}}=\frac{d}{c}$$

*T_pc_SISO_* is given by:

(25) $$T_{pc\_\text{SISO}}=T_{\text{Mod}}+T_{\text{Dmo}d}$$

## 6. Evaluation Results

Simulations were carried out to investigate BER performance *vs.* bit-energy to noise-spectral density ratio *E_b_*/*N_o_* of 16-DQPSK, 16-QAM, 16-OQPSK, 16-FOFDM (without cyclic prefix), 16-WOFDM (4-level symmetric with Haar filter coefficients), BPSK-16FOFDM, and BPSK-16WOFDM with four DSNs (as one transmitting Virtual-MIMO node) and one DGN with three DANs (as one receiving Virtual-MIMO node) using Matlab/Simulink. Therefore, there are eight nodes in total in the system each with a single antenna. Matlab/Simulink is used as the simulation platform as it is one of the most widely used tools for physical layer modeling of wireless systems with many digital communication blocks and analyzing tools available for evaluating system performance. In addition, C and high definition languages (HDL) can be generated directly from Matlab/Simulink code for real hardware implementation.

The information source of each DSN generates data at a rate of 250 kbps according to IEEE 802.15.4-2009 standard for WSNs. The typical transmission range of IEEE 802.15.4 based radio transceivers is 10–20 m, with a nominal maximum range of about 100 m in clear line-of-scenarios. Accordingly, the distance *d^L^* between transmitting and receiving clusters is set to 20 m in this paper.

At each DSN, information bits are modulated into a symbol stream using 16-DQPSK, 16-QAM, 16-OQPSK, 16-FOFDM, 16-WOFDM, BPSK-16FOFDM, and BPSK-16WOFDM. As in [35], the channel response matrix *H* is assumed to be known at DGN to detect the received signals using QR decomposition detection algorithm. All performance graphs are plotted with their 95% confidence intervals.

**Figure 7.**
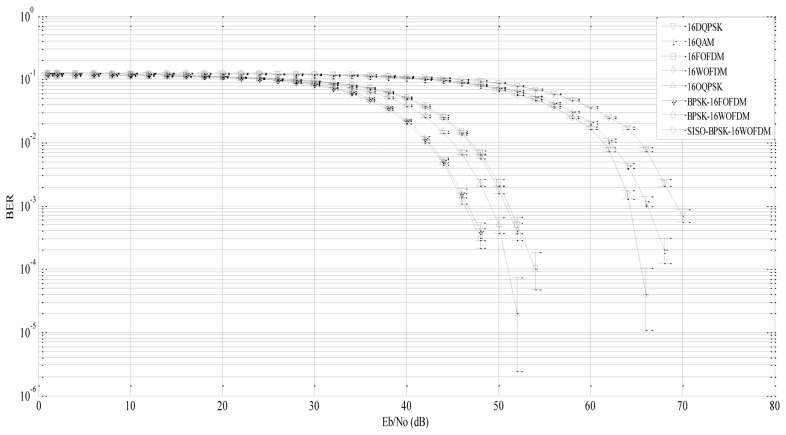
BER performance over transmission distance *d^L^* = 20 m.

Figure 7 shows that FOFDM, WOFDM (including BPSK-FOFDM and BPSK-WOFDM) and OQPSK based systems have better BER performance than QAM and DQPSK because as the constellation set size (*M* = 2*^b^* where *b* is the number of bits) increases, *M*-ary signaling performance improves. BER performance between 16-FOFDM and 16-WOFDM, and that between BPSK-16FOFDM and BPSK-16WOFDM are found to be comparable due to their equivalent filter bank structures [9]. The pair BPSK-16FOFDM and BPSK-16WOFDM is also found to perform better than 16-FOFDM and 16WOFDM. This is because by using BPSK before 16-WOFDM or 16-FOFDM, the Euclidean distance among the signal vectors in the signal space increases due to which the signal energy associated with that distance also increases. Due to the same reason 16QAM performs better than 16DQPSK. BPSK-16WOFDM with SISO architecture expectedly performs worse as compared to BPSK-16WOFDM with virtual MIMO architecture.

**Figure 8.**
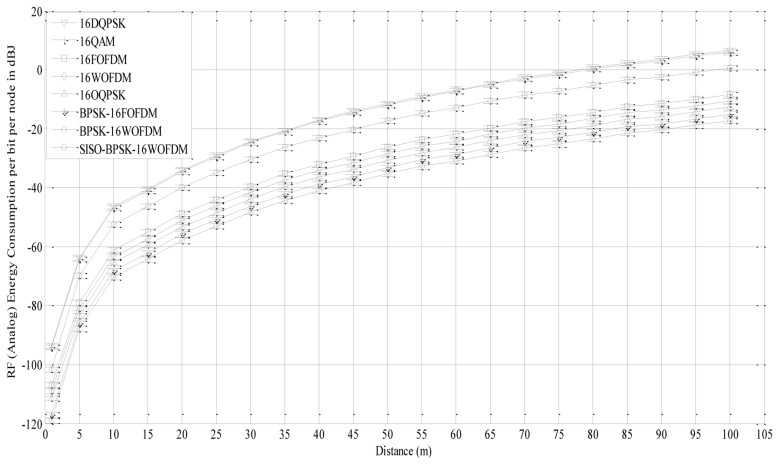
Total energy per bit per node over transmission distance *d^L^* = 1 to 100 m.

The RF (Analog) energy consumption per bit per node over a transmission distance *d^L^* = 1 – 100 m is shown in Figure 8 for a system with four DSNs, three DANs and one DGN each with single antenna using 16-DQPSK, 16-QAM, 16-FOFDM, 16-WOFDM, 16-OQPSK, BPSK-16FOFDM, and BPSK-16WOFDM with a PAPR of 0, 2.55, 3.01, 3.01, 0, 0 and 0 dB respectively. The results are also compared with that of BPSK-16WOFDM with SISO architecture.

In all the simulations, it is assumed *G_t_* = *G_r_* = 8 dBi, *f_c_* = 2.5 GHz, ${\bar{P}}_{b}^{L}={\bar{P}}_{b}^{l}=10^{-3}$, *M_l_* = 40 dB, *N_f_* = 10 dB, *ς* = 0.35, *σ*^2^ = −174 dBm/Hz, *G_ti_* = *G_ri_* = 2 dBi, *d^l^* = 1 *m*, *P_DAC_* = 15.5 mW, *P_ADC_* = 9.8 mW, *P_fil_* = 1.25 mW, *P_LO_* = 50 mW, *P_LNA_* = 20 mW, *P_IFA_* = 3 mW, *P_mix_* = 30.3 mW, *P_Add_* = 10 mW, and *P_PS_* = 5 mW.

The RF (Analog) energy per bit per node is calculated using Equations (9)–(15) for every 5 m of distance to evaluate the effect of distance on energy consumption. It is observed that 16-QAM and 16-DQPSK based systems are the least energy-efficient due to their poor BER performance and complex RF architecture, with the former being the more dominant factor. However, both techniques performed almost the same even though the BER performance of 16-DQPSK is poorer as compared to 16-QAM. This is due to the lower PAPR of 16-DQPSK which resulted in the RF (Analog) energy consumption performance of both techniques to be almost alike. 16-OQPSK system with complex RF architecture performs better than 16WOFDM and 16FOFDM system due to its better BER performance and lower PAPR. 16-WOFDM based system consumes less energy as compared to 16-FOFDM by approximately 40% due to its simpler RF architecture, which reduces the amount of circuit energy it consumes. For the same reason, BPSK-16WOFDM is also found to consume less energy than BPSK-16FOFDM by a similar margin. It is also observed that BPSK-16FOFDM and BPSK-16WOFDM are more energy efficient than 16-FOFDM, and 16-WOFDM, respectively, mainly due to their lower PAPR. From Figure 8, it is clear that virtual MIMO system is more energy efficient as compared to SISO system due to better BER performance.

The base band energy consumption per bit per node (*E_b_Digital_*) for all modulation types along with their constituting energy consumed by individual modulator, demodulator, and detection algorithm are shown in Table 1. It is observed that each modulator consumes more energy as compared to demodulator due to its higher computational complexity (in terms of CPU cycles per bit). For similar reasons, 16-DQPSK consumes less *E_b_Digital_* as compared to other modulation techniques. Since SISO system does not need to perform V-BLAST detection on receiver side (hence *E_Det_* is negligible), the *E_b_Digital_* of BPSK-16WOFDM with virtual MIMO is higher as compared to that of the SISO system.

Table 2 shows the RF (Analog) energy consumption (*E_b__Analog__*) at different transmission distances values and total energy consumption (*E_b_Total__* = *E_b__Analog__* + *E_b__Digital__*). It is clear from Section 5.1.2 that *E_b__Digital__* is independent of transmission distance. For short distance (*d^L^* ≤ 25*m*), *E_b__Digital__* has significant effect on *E_b_Total__*,. However, as *d^L^* increases, *E_b__Analog__* increases, which reduces the effect of *E_b__Digital__*. 16-WOFDM performs better for short distance (*d^L^* ≤ 25*m*) as compared to BPSK-16FOFDM, 16FOFDM, 16-OQPSK and BPSK-16WOFDM as a result of a lower *E_b__Digital__*. For larger distances BPSK-16WOFDM performs better because of a lower *E_b__Analog__*. For all transmission distances, *E_b_Analog_* and *E_b_Total_* of SISO system with BPSK-16WOFDM are higher as compared to virtual MIMO system with BPSK-16WOFDM.

As discussed, the base band (digital) energy consumption and RF (analog) energy consumption is the energy consumed by the CPU, and radio transceiver, of the sensor nodes, respectively. We assume that both the CPU and radio transceiver has two active states (Transmit and Receive). For the CPU, the energy consumption in Transmit mode is the base band energy consumed by the digital modulator (hence depends on the modulation type) for processing each bit for transmission. On the other hand, the CPU or processing energy consumption in Receive mode is the base band energy consumed by the digital demodulator and V-BLAST detection algorithm.

The overall energy consumption per bit per node in Transmit mode (*E_b_Total_Tx_*) and Receive mode (*E_b_Total_Rx_*) for *d^L^* = 20 *m* are shown in Table 3. *E_b_Total_Tx_* is the sum of *E_Mod_* and *E_b_Analog_Tx_* while *E_b_Total_Rx_* is the sum of *E_Dmod_*, *E_Det_* and *E_b_Analog_Rx_*, where *E_b_Analog_Tx_* and *E_b_Analog_Rx_* refers to the RF (analog) energy consumption by the transmitter circuits, and receiver circuits, of the radio transceiver, respectively, shown in Figures 5 and 6.

The CPU is also assumed to have two inactive states (Idle and Sleep), which are low power modes during which different functions of the CPU are shutdown to save power. To calculate the CPU energy consumption in these modes, we used the voltage and current values for these modes given in the data sheet of MSP430F1611 (the CPU model of TelosB mote). The CPU energy consumption per second in idle and sleep mode is found to be −39.5860 dBJ, and −56.1618 dBJ, respectively.

For the radio transceiver, we assume that whenever it is not transmitting or receiving, it will be put into sleep, *i.e.*, it has sleep mode as its only inactive state. The energy consumed by the transceiver in sleep mode will depend on the selected radio components that remain on during sleep state, which is design-specific. However, for most existing transceivers for WSNs, the *sleep*-*to*-*receive* energy consumption ratio is about 0.001, *i.e.*, the energy consumed in sleep mode is typically about 0.1% of the energy consumed in receive mode. Thus, the transceiver's energy consumption per second in sleep mode can be calculated from *E_b_Analog_Rx_* (energy consumption by receiver circuits of the RF section per bit per node), which is found to be −34.8161 dBJ for 16-DQPSK, 16-QAM, 16-OQPSK, 16-FOFDM, and BPSK-16FOFDM, and −36.3601 dBJ for 16-WOFDM and BPSK-16WOFDM.

The time delays involved during the communication are listed in Table 4. It is observed that *T_tr_MIMO_* is 1.75 μs/bit for all modulation techniques. *T_pr_MIMO_* is calculated using Equation (20) for *d^L^* = 10 *m* and *d^L^* = 100 *m*. It is also observed that the modulator incurred a higher processing time (*T_Mod_*) than the demodulator (*T_Dmod_*). This is because more mathematical operations are involved in modulating the signal than demodulating. The total processing delay *T_pc_MIMO_* is considerably high as compared to *T_tr_MIMO_* and *T_pr_MIMO_* due to lower processing speed of TelosB mote. Thus, *T_pc_MIMO_* is the most dominant time delay factor for the total time delay of virtual-MIMO (*T_V_MIMO_*). 16-DQPSK based system is found to incur the least total time delay due to its lower *T_pc_MIMO_* as compared to other six modulation techniques, followed by 16-QAM, 16-WOFDM, BPSK-16WOFDM, 16-OQPSK, 16-FOFDM, and BPSK-16FOFDM. *T_SISO_* is lower than *T_V_*_–_*_MIMO_* because no *T_Det_* is involved in the SISO system. From a comparison of the results between Tables 1 and 4, it can be observed that modulation techniques with less *T_pc_MIMO_* will also exhibit less *E_b_Digital_* value, and vice versa. For example, the *T_pc_MINMO_* and *E_b_Digital_* of 16-DQPSK is 0.2195 s, and −31.2591 dBJ (or 0.7483 × 10^−3^ J), respectively, while for BPSK-16FOFDM, it is 0.2836 s, and −29.8167dBJ (or 1.0431 × 10^−3^ J), respectively.

**Figure 9.**
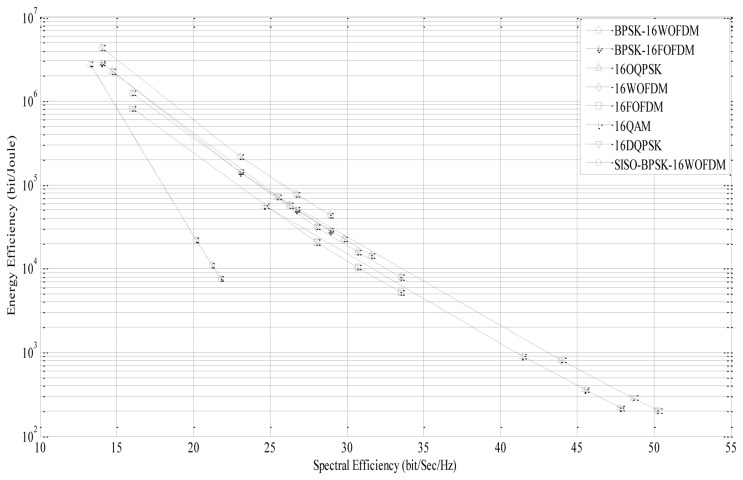
Energy efficiency *vs.* Spectral efficiency over transmission distance *d^L^* = 20 m.

The spectral efficiency can be calculated using Equation (17) for various $\frac{E_{b}}{N_{o}}$ values. The four data points on each curve are obtained by setting the BER values to 10^−1^, 10^−2^, 10^−3^, and 10^−4^. It can be observed from Equation (17) that by increasing $\frac{E_{b}}{N_{o}}$, the spectral efficiency will increase. For a given BER = 10^−3^ (third data point), BPSK-16WOFDM and BPSK-16FOFDM have a spectral efficiency of 27 bit/sec/Hz at $\frac{E_{b}}{N_{o}}=46\;dB$, 16WOFDM and 16FOFDM have a spectral efficiency of 30.7 bit/sec/Hz at $\frac{E_{b}}{N_{o}}=51\;dB$, and 16-QAM has a spectral efficiency of 45.5 bit/sec/Hz at $\frac{E_{b}}{N_{o}}=66\;dB$. The energy efficiency (EE) *versus* spectral efficiency (SE) graph for different modulation techniques is shown in Figure 9. The four data points on each curve are obtained by setting the BER values to 10^−1^, 10^−2^, 10^−3^ and 10^−4^. From the graph, it can be observed that there is a trade-off between EE and SE, where an increase in SE due to higher $\frac{E_{b}}{N_{o}}$ decreases the EE. For a given SE value, it is observed that BPSK-16WOFDM is the most energy efficient technique. In comparison to SISO systems, MIMO systems are more spectrally efficient due to effective bandwidth utilization [36].

## 7. Conclusions

This paper analyzes the performance of a cooperative virtual MIMO system using different modulation techniques in the context of WSNs. In terms of BER performance, BPSK-16WOFDM is found to outperform other evaluated modulation techniques by up to 95% for a given $\frac{E_{b}}{N_{o}}$, and in terms of energy efficiency by up to a factor of two for a transmission distance *d^L^* = 100 m. On the other hand, DQPSK based system performs better in terms of total time delay by up to almost 23%. Thus, DQPSK based system can be a suitable option for WSN applications with less time delay requirement. Virtual MIMO system is 98% more energy efficient as compared to SISO system, which performs better in terms of total time delay by 35%. Overall, BPSK-WOFDM when combined with a cooperative virtual MIMO system architecture shows great potential as a solution for WSNs due to its simpler RF section, lower PAPR and better BER performance.

## Figures and Tables

**Figure 1.**
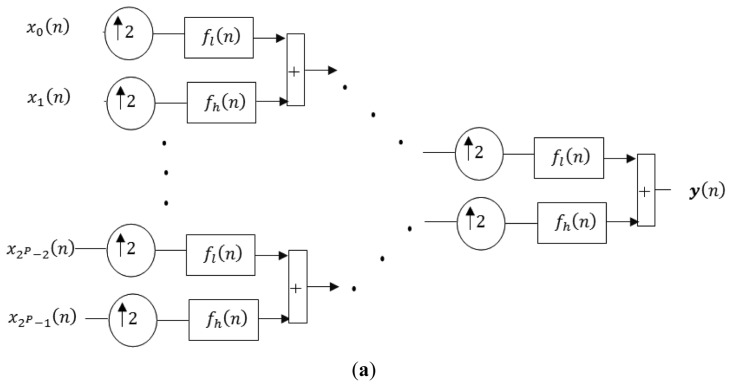

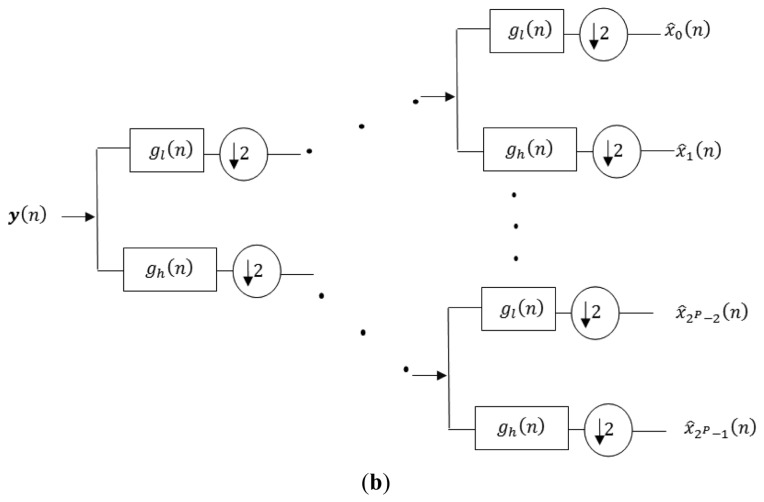
Symmetric multistage WOFDM modulator and demodulator. (**a**) Symmetric multistage synthesis side QMF bank; (**b**) Symmetric multistage analysis side QMF bank.

**Table 1.** Base Band (Digital) Energy Consumption.

**Modulation Type**	***E_Mod_* per bit in dBJ**	***E_Dmod_* per bit in dBJ**	***E_Det_* per bit in dBJ**	***E_b_Digital_* per bit per node in dBJ**
16-DQPSK	−31.7203	−32.1389	−30.7033	−31.2591
16-QAM	−31.2628	−31.9686	−30.7033	−30.9803
16-OQPSK	−30.8951	−31.1203	−30.7033	−30.4600
16-FOFDM	−30.0134	−30.7192	−30.7033	−29.8781
16-WOFDM	−31.0294	−31.7203	−30.7033	−30.8009
BPSK-16FOFDM	−29.86	−30.587	−30.7033	−29.8167
BPSK-16WOFDM	−30.8764	−31.2681	−30.7033	−30.5142
SISO-BPSK-16WOFDM	−30.8764	−31.2681	−∞	−31.0678

**Table 2.** RF (Analog) Energy Consumption and Total Energy Consumption.

**Modulation Type**	***E****_b_Analog_* **per bit per node in dBJ**				***E_b_Total_***=***E_b_Analog_***+ ***E_b_Digital_*** **per bit per node in dBJ**			

	*d^L^* = 25*m*	*d^L^* = 50*m*	*d^L^* = 75*m*	*d^L^* = 100*m*	*d^L^* = 25*m*	*d^L^* = 50*m*	*d^L^* = 75*m*	*d^L^* = 100*m*
16-DQPSK	−29.0354	−11.4148	−1.1630	6.4758	−26.9961	−11.3700	−1.1587	6.4765
16-QAM	−29.5144	−11.8938	−1.6419	5.9967	−27.1754	−11.8405	−1.6369	5.99766
16-OQPSK	−48.4654	−30.6948	−20.6230	−12.6442	−30.3918	−27.5655	−20.1940	−12.5729
16-FOFDM	−44.0580	−26.2874	−16.2156	−8.2368	−29.7153	−24.7115	−16.0327	−8.20722
16-WOFDM	−46.0988	−28.4682	−18.4363	−10.4776	−30.6746	−26.4695	−18.1914	−10.4374
BPSK-16FOFDM	−50.7654	−33.2648	−22.9930	−15.1242	−29.7819	−28.1969	−22.1730	−14.9792
BPSK-16WOFDM	−52.9461	−35.2855	−25.2637	−17.2949	−30.4894	−29.2648	−24.1293	−17.09279
SISO-BPSK-16WOEDM	−30.402	−17.285	−7.263	0.705	−27.7118	−17.107	−7.2449	1.1769

**Table 3.** Energy Consumption in Transmit and Receive Modes.

***Modulation Type***	**Transmit (T_x_) Mode**			**Receive (R_x_) Mode**			

	***E****_Mod_per bit per node in dBJ*	***E****_b_Analog_Tx_per bit per node in dBJ*	***E****_b_Total_Tx_per bit per node in dBJ*	***E****_Dmod_per bit per node in dBJ*	***E****_Det_per bit per node in dBJ*	***E****_b_Analog_Rx_per bit per node in dBJ*	***E****_b_Total_Rx_per bit per node in dBJ*
16-DQPSK	−31.7203	−36.4958	−30.4720	−32.1389	−30.7033	−58.7955	−28.3517
16-QAM	−31.2628	−38.995	−30.5863	−31.9686	−30.7033	−58.7955	−28.2797
16-OQPSK	−30.8951	−55.8558	−30.8813	−31.1203	−30.7033	−58.7955	−27.8965
16-FOFDM	−30.0134	−53.855	−29.9955	−30.7192	−30.7033	−58.7955	−27.7009
16-WOFDM	−31.0294	−56.370999	−31.0167	−31.7203	−30.7033	−60.3395	−28.1718
BPSK-16FOFDM	−29.86	−58.1958	−29.8536	−30.587	−30.7033	−58.7955	−27.6345
BPSK-16WOFDM	−30.8764	−60.741	−30.8719	−31.2681	−30.7033	−60.3395	−27.9662

**Table 4.** Time Delay.

**Modulation Type**	*T_tr_MIMO_***s/bit**	*T_pr_MIMO_***s/bit**		*T_pc_MIMO_***s/bit**			*T_V_*_−_*_MIMO_***s/bit**		

		*d^L^* = 10*m*	*d^L^* = 100*m*	*T_Mod_*	*T_Dmod_*	*T_Det_*	*T_pc_*	*d^L^* = 10*m*	*d^L^* = 100*m*
16-DQPSK	1.75 × 10^−6^	3.33 × 10^−8^	3.33 × 10^−7^	0.069230	0.06286	0.0875	0.21959	0.21959	0.21959
16-QAM	1.75 × 10^−6^	3.33 × 10^−8^	3.33 × 10^−7^	0.076925	0.06537	0.0875	0.2298	0.2298	0.2298
16-OQPSK	1.75 × 10^−6^	3.33 × 10^−8^	3.33 × 10^−7^	0.083717	0.07948	0.0875	0.25070	0.25070	0.25070
16-FOFDM	1.75 × 10^−6^	3.33 × 10^−8^	3.33 × 10^−7^	0.102575	0.08717	0.0875	0.27725	0.27725	0.27725
16-WOFDM	1.75 × 10^−6^	3.33 × 10^−8^	3.33 × 10^−7^	0.0800	0.06922	0.0875	0.236725	0.236727	0.236727
BPSK-16FOFDM	1.75 × 10^−6^	3.33 × 10^−8^	3.33 × 10^−7^	0.10625	0.08987	0.0875	0.28360	0.28360	0.28360
BPSK-16WOFDM	1.75 − 10^−6^	3.33 × 10^−8^	3.33 × 10^−7^	0.084075	0.07682	0.0875	0.24840	0.24840	0.24840

SISO-BPSK-16WOFDM	*T_tr_SISO_* s/bit	*T_pr_SISO_* s/bit		*T_pc_SISO_* s/bit				*T_SISO_* s/bit	

		*d* = 10*m*	*d* = 100*m*	*T_Mod_*	*T_Dmod_*		*T_pc_SISO_*	*d* = 10*m*	*d* = 100*m*

	1 × l0^−6^	3.33 × **10^−8^**	3.33 × **10^−7^**	0.084075	0.07682		0.160895	0.160895	0.160895
